# Empirical voriconazole therapy for febrile neutropenic patients with hematological disorders: a prospective multicenter trial in Japan

**DOI:** 10.1007/s10156-013-0634-5

**Published:** 2013-06-28

**Authors:** Hideo Koh, Masayuki Hino, Kensuke Ohta, Masaki Iino, Fumiaki Urase, Masaki Yamaguchi, Jun Yamanouchi, Noriko Usui, Minoru Yoshida, Mitsune Tanimoto, Kazuma Ohyashiki, Akio Urabe, Kazuo Tamura, Akihisa Kanamaru, Tohru Masaoka

**Affiliations:** 1 Hematology, Graduate School of Medicine, Osaka City University, 1-4-3 Asahi-machi, Abeno-ku, Osaka, 545-8585 Japan; 2 Department of Hematology, Osaka Saiseikai Nakatsu Hospital, Osaka, Japan; 3 Medical Oncology, Yamanashi Prefectural Central Hospital, Yamanashi, Japan; 4 Department of Hematology, Sakai Hospital Kinki University Faculty of Medicine, Osaka, Japan; 5 Department of Hematology, Ishikawa Prefectural Central Hospital, Ishikawa, Japan; 6 Department of Bioregulatory Medicine, Ehime University Graduate School of Medicine, Ehime, Japan; 7 Division of Clinical Oncology and Hematology, Department of Internal Medicine, The Jikei University School of Medicine, Tokyo, Japan; 8 Voriconazole Study Group for Febrile Neutropenia, Osaka, Japan

**Keywords:** Prospective multicenter study, Voriconazole, Empirical antifungal therapy

## Abstract

An open-label, prospective, multicenter study was conducted between October 2006 and March 2010 to assess the efficacy and safety of intravenous voriconazole (VRCZ) as empirical therapy for antibiotic-refractory febrile neutropenia in Japanese patients with hematological disorders. In addition, to find the patient groups that may benefit from antifungal therapy, the definition of invasive fungal infection proposed by EORTC/MSG (2002) was assessed in this study. Plasma (1-3)-β-d-glucan and *Aspergillus* PCR in blood were also measured to improve the diagnostic accuracy. A total of 103 patients (median age, 59 years), including 25 undergoing induction chemotherapies and 19 allogeneic hematopoietic cell transplants, were evaluable. Sixty-nine percent of the patients achieved resolution of clinical symptoms and 31 % achieved treatment success, defined as fulfilling the previously described five-part composite endpoint. Although VRCZ was discontinued in 9.7 % of the patients because of adverse effects, all the patients recovered soon after discontinuation of VRCZ. The treatment success rate of VRCZ appeared to be higher in patients categorized as “not classified” compared with “possible invasive fungal disease” according to the EORTC/MSG criteria. Moreover, six “not classified” patients were positive for either plasma (1-3)-β-d-glucan (*n* = 5) or *Aspergillus* PCR in blood (*n* = 2). The present study demonstrates that empirical VRCZ therapy is safe and effective in Japanese patients. Additionally, (1-3)-β-d-glucan and *Aspergillus* PCR tests were expected to provide additional information on the diagnosis of invasive fungal infections.

## Introduction

It is well known that invasive fungal infections (IFIs) cause significant morbidity and mortality in neutropenic patients with hematological malignancies who have undergone intensive chemotherapy or received hematopoietic stem cell transplantation [1–4]. Because an early diagnosis of IFIs remains quite difficult while a delay in antifungal therapy increases mortality, empirical antifungal therapy, triggered by persistent fever refractory to broad-spectrum antibacterial therapy, has been the standard of care for many decades [5, 6].

Voriconazole (VRCZ), a fluconazole-derived new triazole, exerts excellent antifungal activity by preventing fungal cell growth via inhibition of fungal cytochrome P450-dependent 14α-lanosterol demethylation, indispensable for the synthesis of ergosterol in fungal cell membranes [7–10]. In a major, multicenter, clinical trial that prospectively examined the efficacy and safety of VRCZ as empirical therapy, VRCZ was shown to be less toxic compared with liposomal amphotericin B but failed to meet the strict statistical noninferiority criteria [11]. Importantly, however, this study strongly suggested that VRCZ reduced the incidence of documented breakthrough invasive aspergillosis, candidemia, and dematiaceous fungal infections, especially in the high-risk group without antifungal prophylaxis compared with liposomal amphotericin B [11]. Moreover, in another randomized trial comparing VRCZ and amphotericin B as target therapy for definite or probable invasive pulmonary aspergillosis, not only the efficacy but also the survival rates were significantly in favor of VRCZ, suggesting the strong antifungal activity of VRCZ in neutropenic patients [10]. Therefore, it is important to confirm the utility of VRCZ as empirical therapy in an additional prospective study, although such a trial has not been performed until now.

However, it is widely accepted that more than half of antibiotic-resistant fevers are not caused by fungal infection; consequently, there is a tradeoff between the benefits of empirical therapies and the adverse effects arising from unnecessary antifungal treatments [12, 13]. Moreover, adequate statistical evaluations in randomized comparative studies for empirical therapy have been hampered by the unavoidable inclusion of patients who do not have any fungal infection. To more effectively target patients who may benefit from antifungal therapy, therefore, various “preemptive” approaches have recently been proposed in which antifungal therapy is withheld until additional clinical, mycological, or radiologic findings suggesting the presence of an IFI are obtained [14, 15]. However, although these preemptive approaches have been shown to reduce the number of antifungal therapies, recent international guidelines concluded that it is still premature at present to introduce the preemptive approach as an alternative to the empirical approach for safety concerns [5, 6]. Therefore, to establish safer preemptive strategies in future, it is necessary to identify the appropriate diagnostic tools and the timing of their use as well as to identify the patients who would benefit from such an approach.

On the basis of these observations, we conducted a prospective multicenter trial to confirm the efficacy of VRCZ as empirical therapy in Japanese patients with hematological diseases. In addition, we analyzed the efficacy of VRCZ in accordance with the definitions proposed by EORTC/MSG [16]. Furthermore, we also examined the usefulness of the plasma (1-3)-β-d-glucan test and *Aspergillus* polymerase chain reaction (PCR) assay in blood [17–20] to improve the diagnostic accuracy.

## Patients and methods

### Study design

The study was an open-label, prospective, multicenter clinical trial of VRCZ, conducted between October 2006 and March 2010 at 21 institutions in Japan. All the institutional review boards and ethics committees of the institutions involved approved the protocol and consent form, and written informed consent was obtained from all patients before enrollment.

### Inclusion and exclusion criteria

Patients aged 16 years or older were included if they had received chemotherapy for hematological disorders or hematopoietic stem cell transplantation and had febrile neutropenia refractory to broad-spectrum antibacterial therapy for 3 days or more. Neutropenia was defined as a neutrophil count <500/μl, or <1,000/μl with the expectation that it would further decrease to <500/μl within a few days. Fever was defined as an axillary temperature ≥37.5 °C or an oral temperature ≥38.0 °C based on a single measurement. Patients were excluded if they had documented IFIs at study entry, severe liver dysfunction (aminotransferase or total bilirubin levels ≥5 times the upper limit of normal), renal failure (serum creatinine ≥2.5 mg/dl), or a history of allergy to VRCZ or other azoles. For study entry, prophylactic antibacterial and antiviral therapies, or previous antifungal administration was permitted according to each institution’s protocol, but therapeutic use of systemic antifungal drugs was not permitted.

### Empirical therapy with VRCZ

As a general rule, VRCZ was administered intravenously, at least for the first 7 days, and switching to oral VRCZ (200 mg twice per day) was permitted thereafter. For patients with specific conditions such as renal dysfunction, however, the use of oral VRCZ from the beginning was permissible. The initial loading dose of intravenous VRCZ was 6 mg/kg twice on the first day, followed by 4 mg/kg twice per day. In cases in which treatment was successful, it was recommended to continue VRCZ for 3 days after achievement of both defervescence and neutrophil recovery, or for at least 14 days in cases of persistent neutropenia. A treatment period of at least 7 days was set, as this was the minimum period required before it could be concluded that treatment had failed.

Before the initiation of VRCZ treatment, serum galactomannan antigen (GM), plasma (1-3)-β-d-glucan, and *Aspergillus* PCR in whole blood were measured together with two sets of blood culture including mycological culture, and a chest X-ray was performed. Chest computed tomography (CT) was recommended but was not mandatory in this study. All measurements of the three microbiological biomarkers were performed at SRL (Tokyo, Japan). Serum GM was measured using a one-stage immunoenzymatic sandwich microplate assay (Platelia *Aspergillus* EIA; cutoff value 0.5, Bio-Rad Laboratories) according to the manufacturer’s instructions and was monitored twice a week. Plasma (1-3)-β-d-glucan was also measured twice a week in accordance with the manufacturer’s protocol (cutoff value, 20 pg/ml for the Fungitec *G* Test; Seikagaku Corporation, Tokyo, Japan). An *Aspergillus* PCR test was also performed at the end of the therapy. The assays were performed with the ABI PRISM 7700 Sequence Detection System (lower limit of detection, 1.0 × 10^2^ copies/ml; Applied Biosystems Japan). In brief, genomic DNA was extracted from whole blood samples and examined by real-time PCR using the TaqMan probe. The sequences of PCR primers were designed to target the highly conserved region of multicopy genes encoding the 18S ribosomal RNA of *Aspergillus* species (*Aspergillus* *fumigatus*, *A.* *terreus*, *A. niger*, *A. flavus*, and *A. nidulans*) [21–23]. The forward primer was 5′-GCGAGTACTGGTCCGGCTGGA-3′, and the reverse primer was 5′-CTAGAAACCAACAAAATAGAACCGC-3′. Although the performance of this real-time PCR system is well established in the clinical setting for some other pathogens including cytomegalovirus and Epstein–Barr virus [24, 25], standardization and validation for *Aspergillus* species have not yet been completed. Therefore, the objectives of using the assay were limited to ascertaining whether it could provide additional information in the diagnosis of invasive *Aspergillosis*.

### Outcome analysis

Patients were not evaluable for efficacy if they had not continued study medication for 7 or more days for a reason other than defervescence, or toxicity or intolerance of VRCZ. Efficacy was evaluated by two outcome measures, clinical efficacy and treatment success. Clinical efficacy was defined as resolution of fever regardless of neutrophil recovery and survival for 7 days after the discontinuation of VRCZ therapy. The definition of treatment success was when the patient met all five criteria of the composite endpoint as follows: (1) survival for at least 7 days after the completion of study drug administration, (2) defervescence for at least 48 h during neutropenia, (3) no breakthrough fungal infection on VRCZ, (4) successful treatment of any baseline fungal infection, and (5) no premature withdrawal of VRCZ because of intolerance or lack of efficacy as reported in previous studies [11, 26, 27]. The baseline fungal infection was defined as documented fungal infection found within the first 48 h after the start of VRCZ administration.

At study entry, patients were classified into “proven,” “probable,” or “possible” IFI according to the criteria proposed by EORTC/MSG in 2002 [16]. In brief, although “proven” IFIs were defined based on definite histopathological or mycological evidence, “probable” and “possible” IFIs were diagnosed based on three factors: host factors, clinical features, and mycological evidence [16]. All the patients enrolled in this study had to have “febrile neutropenia refractory to broad-spectrum antibiotics” as a host factor. Therefore, the patients who either had clinical features or for whom mycological evidence was found were diagnosed as “possible” IFI, and those that had both the clinical feature and mycological evidence were diagnosed as “probable” IFI. Among the detailed descriptions found in the original literature [16], pulmonary lesions or symptoms are commonly observed clinical features, including major criteria (halo sign, air-crescent sign, cavity) and minor criteria (lower respiratory symptoms, pleural rub, nonspecific infiltrate). In these cases, according to the literature, the presence of one major or two minor criteria was regarded as a clinical feature. On the other hand, positive GM, but not positive (1-3)-β-d-glucan and *Aspergillus* PCR tests, was regarded as mycological evidence.

The data review committee, composed of independent experts from the Voriconazole Study Group for Febrile Neutropenia, reviewed the validity of inclusion of all participants, diagnosis, efficacy data, and safety data. No changes to the study were made on the basis of this review.

### Safety

Safety was assessed in all patients who received at least one dose of VRCZ. Investigators followed up adverse events prospectively. Laboratory tests were performed at study entry, as appropriate during therapy, and 1 week after completion of therapy. Severe liver toxicity was defined as >5 times the upper limit of normal of amino transferases if ≤2 times the upper limit of normal at baseline; or >10 times the upper limit of normal of amino transferases if >2 times the upper limit of normal at baseline. Severe nephrotoxicity was defined as >2 times the baseline level of serum creatinine if ≤1.5 mg/dl at baseline; or >3 mg/dl if >1.5 mg/dl at baseline.

### Statistical analysis

Clinical efficacy and treatment success rates were compared between categories by Fisher’s exact test. For comparison of two groups of data that were not normally distributed, the Mann–Whitney *U* test was used. All *P* values were two tailed. All statistical analyses were performed using PASW Statistics, version 17.0 (SPSS, Chicago, IL, USA).

## Results

### Patients

A total of 117 patients were enrolled in this study. Of these, we excluded 11 participants for whom information about outcomes was missing. We also excluded 3 participants because the study medication was discontinued before the elapse of 7 days even though there were no adverse events attributable to the study drug or other definite causes. Thus, the analytic cohort consisted of 103 patients. The baseline characteristics of the study participants are shown in Table 1. The median age of the patients (66 % were male) was 59 years (range, 18–79 years). The major underlying diseases were acute leukemia (67.9 %) and non-Hodgkin lymphoma (15.5 %). Before study entry, 26 patients (25.2 %) had received induction chemotherapy, 2 patients (1.9 %) had undergone autologous hematopoietic stem cell transplantation, and 19 (18.4 %) had received allogeneic hematopoietic stem cell transplantation. According to the criteria proposed by EORTC/MSG in 2002, “proven” IFI, “probable” IFI, “possible” IFI, or “not classified” was found in 0 (0.0 %), 1 (1.0 %), 13 (12.6 %), and 89 (86.4 %) patients, respectively. The median duration of therapy was 11 days (range, 2–37).

**Table 1:** Baseline characteristics of study participants

Characteristic	Total (*n* = 103)
Gender, *n* (%)	
Male	68 (66.0)
Female	35 (34.0)
Median age (range), (years)	59 (18–79)
Underlying disease, *n* (%)	
Acute myeloid leukemia	57 (55.3)
Acute lymphoblastic leukemia	13 (12.6)
Chronic myeloid leukemia	3 (2.9)
Myelodysplastic syndrome/acute myeloid leukemia	10 (9.7)
Non-Hodgkin lymphoma	16 (15.5)
Adult T-cell leukemia/lymphoma	3 (2.9)
Aplastic anemia	1 (1.0)
ECOG performance status	
0	6 (5.8)
1	42 (40.8)
2	31 (30.1)
3	22 (21.4)
4	2 (1.9)
Treatment of hematological disease, *n* (%)	
Chemotherapy	80 (77.7)
Induction	26 (25.2)
Consolidation	14 (13.6)
Others	40 (38.8)
Autologus hematopoietic stem cell transplantation	2 (1.9)
Allogeneic hematopoietic stem cell transplantation	19 (18.4)
Missing	2 (1.9)
Classification according to EORTC/MSG 2002 criteria, *n* (%)	
Not classified	89 (86.4)
Possible	13 (12.6)
Probable	1 (1.0)
Proven	0 (0.0)
Chest computed tomography examination, *n* (%)	49 (47.6)
Neutrophil count at study entry (/μl), median (range)	2 (0–4,936)
Duration of fever before the start of voriconazole administration (days), median (range)^a^	5 (2–22)
Duration of administration of voriconazole (days), median (range)	11 (2–37)
Route of administration	
Intravenous	96 (93.2)
Oral	7 (6.8)

^a^Two cases were not included because information was missing

### Efficacy

Clinical efficacy was demonstrated in 68.9 % of patients whereas the treatment success rate according to the criteria of the five-part composite endpoint was 31.1 % (Table 2). Neither baseline nor breakthrough fungal infections were observed in the present study. Based on the clinical decision made at the participating institutions, seven patients were treated with oral VRCZ from the beginning. In these patients, renal dysfunction was not severe, with a median serum creatinine level of 1.08 (0.70–1.34) mg/dl, and no other specific information on clinical background was available. The clinical efficacy and treatment success rates for these patients were 57.1 % (4/7) and 14.3 % (1/7), respectively.

**Table 2:** Overall efficacy

Outcome measure	*n*/*n* (%)
Clinical efficacy	71/103 (68.9)
Treatment success using composite endpoint^a^	32/103 (31.1)
Survival for at least 7 days after completion of study drug administration	98/103 (95.1)
Deferverscence during neutropenia	33/103 (32.0)
No breakthrough fungal infection	103/103 (100.0)
No premature withdrawal of voriconazole because of intolerance or lack of efficacy	86/103 (83.5)

^a^A baseline fungal infection was not observed in this study

We further analyzed the differences in efficacy of VRCZ by patient subgroups categorized according to the types of treatment for hematological diseases, EORTC/MSG criteria, neutrophil counts at study entry, and duration of fever before the start of VRCZ administration. As shown in Table 3, no significant difference in efficacy was observed among the subgroups categorized by type of treatment for underlying diseases and duration of fever before the start of VRCZ administration. However, both clinical efficacy and treatment success rates tended to be lower in the “possible” subgroup compared with the “not classified” subgroup according to the EORTC/MSG criteria. When the patients were categorized according to neutrophil count at the start of VRCZ administration, the treatment success rate, but not clinical efficacy, was significantly higher in the groups having lower neutrophil counts. To ascertain the reasons for these observations, we further evaluated the duration of neutropenia after the start of VRCZ administration in patients where treatment was clinically effective. Among the 62 evaluable patients (neutrophil count at the start of VRCZ <500/μl and recovered thereafter), the duration of neutropenia in the subgroup having an initial neutrophil count of <100/μl was 6 days (range, 2–24) whereas that having an initial count from ≥100/μl to <500 μl was 3 days (range, 1–13) (*P* < 0.001). These results make it clear that when the duration of neutropenia is shorter after the start of VRCZ administration, the probability of satisfying “resolution of fever during neutropenia” (one of the components of the composite endpoint) is lower, as also discussed elsewhere [28]. Therefore, underestimation of efficacy was considered to be at least partially responsible for the lower treatment success rate in higher neutrophil subgroups (≥100/μl).

**Table 3:** Clinical efficacy and treatment success rate according to treatment of hematological diseases, EORTC/MSG 2002 criteria, neutrophil count at study entry, or duration of fever before treatment with voriconazole

	Clinical efficacy, *n*/*n* (%)	*P*	Treatment success, *n*/*n* (%)	*P*
Treatment of hematological diseases^a^		0.466		0.466
Chemotherapy	54/80 (67.5)		27/80 (33.8)	
Autologus hematopoietic stem cell transplantation	1/2 (50.0)		0/2 (0.0)	
Allogeneic hematopoietic stem cell transplantation	15/19 (78.9)		4/19 (21.1)	
Classification according to EORTC/MSG (2002) criteria		0.021		0.462
Not classified	65/89 (73.0)		30/89 (33.7)	
Possible	5/13 (38.5)		2/13 (15.4)	
Probable	1/1 (100.0)		0/1 (0.0)	
Neutrophil count at study entry (/μl)		0.708		0.025
<100	50/72 (69.4)		28/72 (38.9)	
100–499	16/25 (64.0)		4/25 (16.0)	
≥500	5/6 (83.3)		0/6 (0.0)	
Duration of fever before treatment with voriconazole (days)^a^		0.828		0.831
≤5 (median value)	37/54 (68.5)		18/54 (33.3)	
>5 (median value)	34/47 (72.3)		14/47 (29.8)	

^a^Two cases were not included because of missing information

### Microbiological study

Thirteen possible IFI cases according to the EORTC/MSG classification were diagnosed based on positive clinical criteria, and 1 case on a positive GM test result (Table 4). One probable IFI case was diagnosed based on positive results for both radiologic findings and GM test. However, 5 of 6 cases with a positive (1-3)-β-d-glucan test were categorized as “not classified” because neither clinical nor mycological criteria were satisfied according to the EORTC/MSG classification (2002). In addition, two cases with a positive *Aspergillus* PCR test were also categorized as “not classified.”

**Table 4:** Number of cases with positive clinical criteria and/or positive on microbiological tests

		Required for EORTC/MSG 2002 criteria			Other indirect blood tests	
	*N*	Clinical criteria^a^	Culture	GM	(1-3)-β-d-glucan	*Aspergillus* PCR
Not classified, *n* (%)	89	0 (0.0)	0 (0.0)	0 (0.0)	5 (5.6)	2 (2.2)
Possible, *n* (%)	13	12 (92.3)	0 (0.0)	1 (7.7)	1 (7.7)	0 (0.0)
Probable, *n* (%)	1	1 (100.0)	0 (0.0)	1 (100.0)	0 (0.0)	0 (0.0)

^a^One major or two minor clinical criteria including radiologic findings or symptoms were needed

Details of the nine patients who were positive for GM, (1-3)-β-d-glucan, or *Aspergillus* PCR are listed in Table 5. One of two patients who had a positive GM test and one of six patients who had a positive (1-3)-β-d-glucan test had nodular lesions with or without halo sign on chest CT, which was strongly suggestive of invasive mold infections. In addition, three other patients who had a positive (1-3)-β-d-glucan test or *Aspergillus* PCR test had nonspecific pulmonary signs or symptoms that met the minor clinical criteria of the EORTC/MSG classification.

**Table 5:** Details of the patients who were positive by galactomannan antigen (GM), (1-3)-β-d-glucan, or *Aspergillus* polymerase chain reaction (PCR) tests

Case no.	GM	(1-3)-β-d-glucan	*Aspergillus* PCR	Chest computed tomography	Clinical symptoms	EORTC/MSG 2002 criteria	Clinical efficacy/treatment success
4	+	–	−	Halo sign	−	Probable	Yes/no
5	+	−	−	Non-specific infiltration	−	Possible	Yes/yes
76	−	+	−	Nodular lesion	−	Possible	Yes/yes
30	−	+	−	Normal	−	Not classified	No/no
81	−	+	−	Normal	−	Not classified	Yes/yes
90	−	+	−	Normal	−	Not classified	No/no
94	−	+	−	Normal	Cough, sputum	Not classified	Yes/no
53	−	+	+	Not done	Chest pain	Not classified	Yes/no
57	−	−	+	Pleural effusion	−	Not classified	Yes/no

### Safety and tolerability

A safety and tolerability analysis was performed for 113 patients (Table 6). Abnormal vision, which was usually transient, was the most common adverse effect, and was observed frequently during the initial infusion. Liver toxicity was the second most common adverse effect of VRCZ. The incidence of drug-related adverse effects was 26.5 %. Treatment with VRCZ was discontinued in 11 patients (9.7 %) because of adverse events including skin eruption and liver dysfunction. However, none of these was fatal, and all patients recovered soon after discontinuation of study drug.

**Table 6:** Drug-related adverse events and causes of VRCZ discontinuation because of adverse events (*n* = 113)

Drug-related adverse events	*n* (%)	Cause of discontinuation	*n* (%)
Total	30 (26.5)		11 (9.7)
Abnormal vision	12 (10.6)	Skin eruption	4 (3.5)
Liver dysfunction	6 (5.3)	Liver dysfunction	2 (1.8)
Skin eruption	4 (3.5)	Abnormal vision	1 (0.9)
Nausea	3 (2.7)	Interstitial pneumonia	1 (0.9)
Interstitial pneumonia	1 (0.9)	Dyspnea	1 (0.9)
Dyspnea	1 (0.9)	Renal dysfunction	1 (0.9)
Renal dysfunction	1 (0.9)	Numbness in lower extremities	1 (0.9)
Numbness in lower extremities	1 (0.9)		
Edema	1 (0.9)		

## Discussion

Because the empirical use of VRCZ in the original report by Walsh et al. failed to satisfy the statistical criteria for noninferiority to liposomal amphotericin B [11], recent guidelines have postulated VRCZ is a safe “alternative” to liposomal amphotericin B [5, 6]. However, this “not non-inferior” result has been debated in a series of subsequent reports, particularly because of the open-label design and the inherent problems rising from the use of the five-part composite endpoint [29, 30]. Moreover, a significantly low rate of breakthrough fungal infections as well as lower toxicity profiles in the VRCZ arm, demonstrated in the same study, suggest that there is no significant disadvantage to the patients when VRCZ is used instead of other established antifungals. Therefore, we are of the opinion that reevaluation of empirical VRCZ in Japanese patients does not raise any ethical concerns, and this position was accepted by our ethical committees. To our knowledge, this is the second report that prospectively evaluated the efficacy of VRCZ using the empirical approach.

The present study demonstrated that VRCZ for empirical therapy is effective and safe in Japanese patients with hematological diseases. However, because the treatment success rate of seven patients who were treated with the oral form of VRCZ seemed to be lower than those who received the intravenous form, oral therapy would not appear to be appropriate except in cases of severe renal dysfunction.

To make an objective comparison with the original report by Walsh et al. [11], we evaluated the efficacy of VRCZ in terms of both “clinical efficacy” (assessed based on the resolution of fever and other clinical symptoms) and “treatment success” using the five-part composite endpoint. The results showed that the treatment success rate in the present study (31.1 %) was at least comparable to that in the original report (26.0 %). Moreover, no documented breakthrough fungal infection was observed in the present study. This finding accorded closely with the findings in the original report in which a low frequency of breakthrough infections was observed in the VRCZ arm, which provides additional evidence of the high level of efficacy of VRCZ in preventing breakthrough IFDs using the empirical approach.

The large discrepancy between the clinical efficacy and treatment success rates in this study can be almost exclusively attributable to the low rate of “resolution of fever during neutropenia” that was included in the composite endpoint. As described in the “Results” section, the achievement rate of this endpoint was particularly low in the patients with higher neutrophil counts at the start of VRCZ. This finding was in good agreement with previous reports suggesting that significant problems are associated with this endpoint [28].

In this study, we further evaluated the relationship between clinical effects of VRCZ and the probability of fungal infection in each patient as defined by the EORTC/MSG 2002 criteria. The results showed that both the clinical efficacy and the treatment success rates of VRCZ were higher in the patients categorized as “not classified” compared with “possible” IFI. However, the latter observation was not statistically significant, possibly because the number of the patients was insufficient (Table 3). The observation was somewhat controversial because the majority of “not classified” patients are expected to have nonfungal fever that theoretically does not respond to VRCZ. The reasons for these observations were not elucidated in the present study. However, some hypotheses may be proposed, including (1) the “not classified” group may include more responders to the antibiotics in combination with VRCZ, (2) the EORTC/MSG criteria may fail to identify early fungal infection, (3) poor availability of the efficacy analysis method, or (4) some other mechanisms that led to the resolution of fevers in the “not classified” group. To gain further insight into these possibilities, improvements in both diagnostic methods and outcome analysis are vital.

As the present study was planned in 2005, we employed the original EORTC/MSG criteria published in 2002, not the revised version published in 2008, which has possibly better diagnostic performance [16, 31–33]. The revised version made a series of important corrections, including making a CT scan mandatory for the diagnosis of pulmonary fungal lesions. However, the present study design did not make a CT scan mandatory, and fewer than half of the patients actually underwent CT. Moreover, the EORTC/MSG 2008 criteria introduced the (1-3)-β-d-glucan test to increase the probability of a correct diagnosis. In addition, although the fungal PCR assay was not adopted in the revised version, it was suggested that this assay should be incorporated in future after the establishment of validated or standardized methods [20, 31]. In our study, six patients diagnosed as “not classified” based on the 2002 criteria were positive for either (1-3)-β-d-glucan (*n* = 5) or *Aspergillus* PCR (*n* = 2) (Table 5). Although this finding suggests that these tools may increase the sensitivity of diagnosis, we could not confirm whether these patients actually had fungal infections.

The present study has several limitations. First, the results may have been affected by selection bias because of the open-label, uncontrolled design of this study. Second, we did not monitor serum VRCZ levels that might have significantly affected the efficacy of VRCZ [34, 35].

In conclusion, the current study demonstrated that VRCZ is a highly potent and safe agent for empirical antifungal therapy in Japanese patients. Additionally, the (1-3)-β-d-glucan and *Aspergillus* PCR tests provided helpful information in the diagnosis of IFIs. Further improvement to diagnostic methods must be considered to establish effective preemptive antifungal therapy.

## Appendix

Investigators who participated in this trial: Saga Medical School Hospital, Saga: Eisaburo Sueoka; Chugoku Central Hospital, Hiroshima: Akira Miyata; Tokai University Hospital, Kanagawa: Kiyoshi Ando; University of Fukui Hospital, Fukui: Katsunori Tai; Rinku General Medical Center, Osaka: Kazuo Hatanaka; Kanazawa Medical University Hospital, Ishikawa: Toshihiro Fukushima; Saga Prefectural Hospital Koseikan, Saga: Nobuo Kuwabara; Tokyo Metropolitan Ohtsuka Hospital, Tokyo: Yasunobu Nagata; Tokushima University Hospital, Tokushima: Shingen Nakamura; Hiroshima University Hospital, Hiroshima: Hideo Hyodo.
